# Supplementary material for: Towards high-throughput parallel imaging and single-cell transcriptomics of microbial eukaryotic plankton
Source: PLoS One. 2024 Jan 19;19(1):e0296672. doi: 10.1371/journal.pone.0296672 (PMC10798536; doi:10.1371/journal.pone.0296672)
Supplement: S1 Fig — Upper and lower columns correspond to the same cells, collected at the same time points. (PDF) [file pone.0296672.s001.pdf]

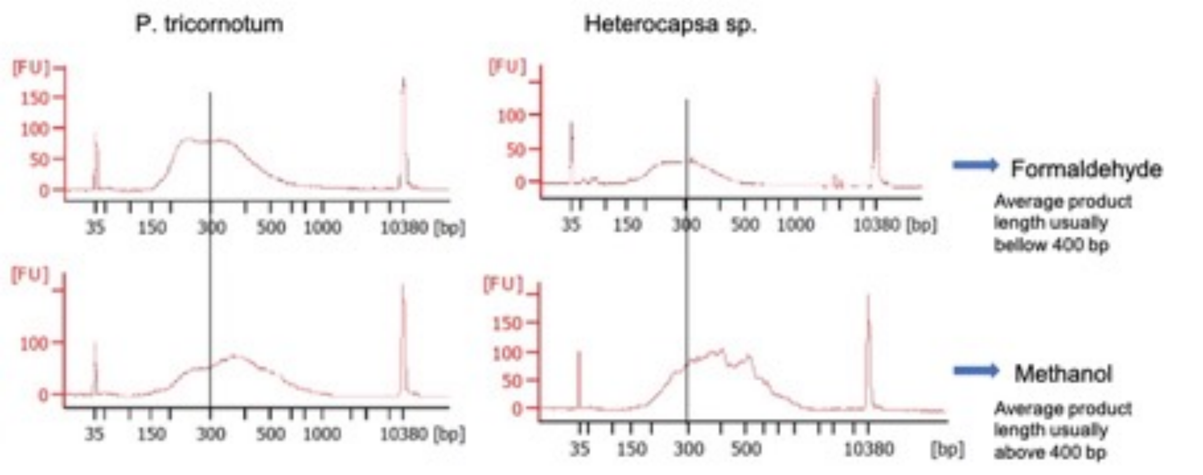
P. tricornotum
Heterocapsa sp.
[FU]
150
100
50
0
35
150
300
500
1000
10380 [bp]
[FU]
100
50
0
35
150
300
500
1000
10380 [bp]
Formaldehyde
Average product length usually bellow 400 bp
[FU]
100
0
35
150
300
500
1000
10380 [bp]
[FU]
150
100
50
0
35
150
300
500
10380
[bp]
Methanol
Average product length usually above 400 bp
