# Supplementary figures and images for: Towards high-throughput parallel imaging and single-cell transcriptomics of microbial eukaryotic plankton
Source: PLoS One. 2024 Jan 19;19(1):e0296672. doi: 10.1371/journal.pone.0296672 (PMC10798536; doi:10.1371/journal.pone.0296672)

## Pre-fixed

*Heterocapsa* spp.

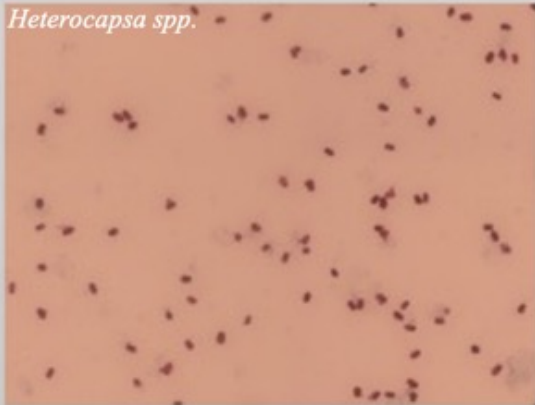

## Fixed on the slide

*Heterocapsa* spp.

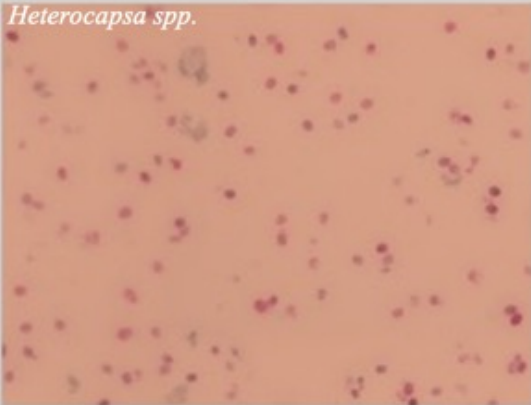

*P. tricornutum*

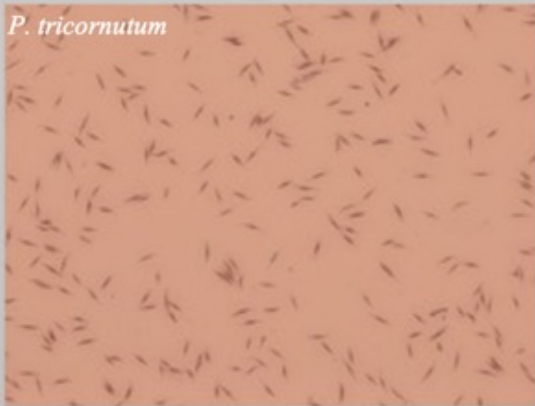

*P. tricornutum*

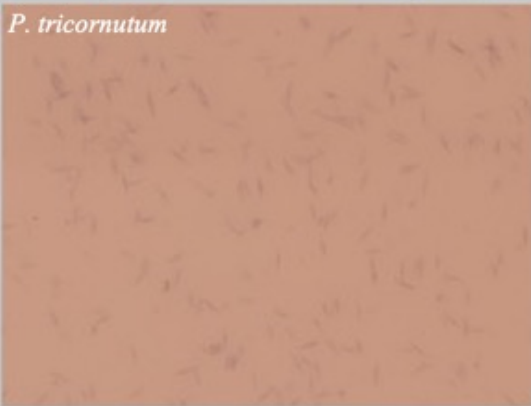

Supplement: S2 Fig — (PDF) [file pone.0296672.s002.pdf]

# Before freeze thaw

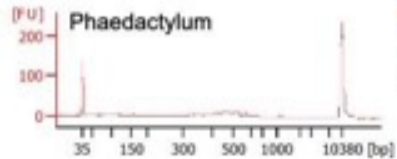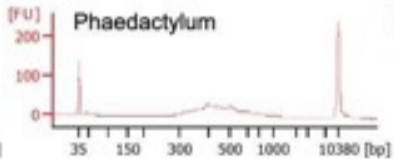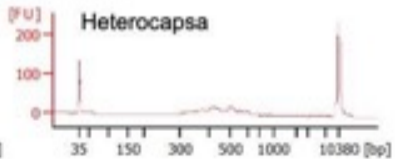

# After freeze thaw

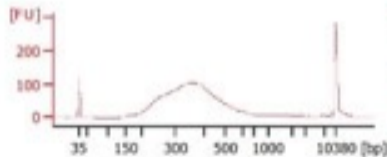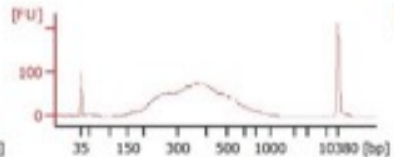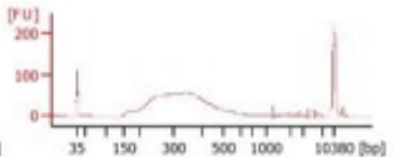

Supplement: S3 Fig — Upper and lower columns correspond to the same cells, collected at the same time points. (PDF) [file pone.0296672.s003.pdf]

A

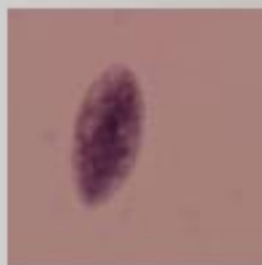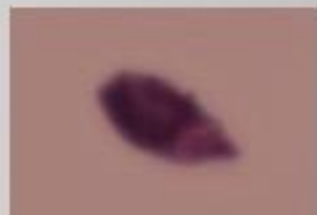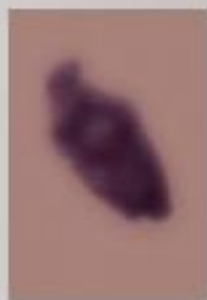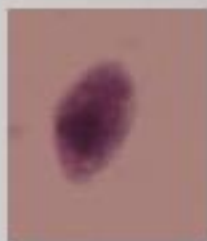

B

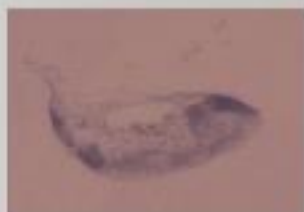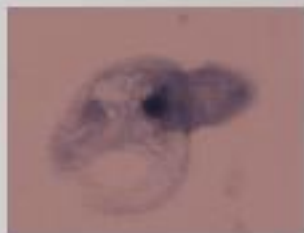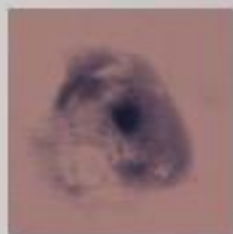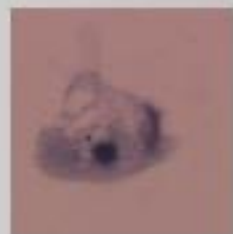

Supplement: S4 Fig — Examples of A) T. thermophila cells prefixed with methanol, attached to the slide and stained with hematoxylin and eosin; B) T. thermophila cells prefixed with methanol and then freeze-thawed in liquid nitrogen and room temperature water (3 times, attached on the slide and stained with hematotoxylin and eosin). (PDF) [file pone.0296672.s004.pdf]
