# Supplementary material for: Towards high-throughput parallel imaging and single-cell transcriptomics of microbial eukaryotic plankton
Source: PLoS One. 2024 Jan 19;19(1):e0296672. doi: 10.1371/journal.pone.0296672 (PMC10798536; doi:10.1371/journal.pone.0296672)
Supplement: S1 Table — (PDF) [file pone.0296672.s005.pdf]

**Reagent**	**Concentration**	**Temperature**	**Time**
Lysozyme solution*	10 mg/ml	37° C	30 min<br>60 min
HCl	0.01M	37° C	10 min<br>20 min
Pepsin#	0.1 g ml^{-1}	37° C	1 min<br>3 min<br>5 min<br>10 min

* To prepare lysozyme solution add 100 mg lysozyme to 1 ml 0,5 M EDTA (pH 8), 1 ml 1M Tris/HCl (pH 8) and 8 ml nuclease free water

Diluted 1:100 in 0.1M HCl
