# Supplementary material for: Towards high-throughput parallel imaging and single-cell transcriptomics of microbial eukaryotic plankton
Source: PLoS One. 2024 Jan 19;19(1):e0296672. doi: 10.1371/journal.pone.0296672 (PMC10798536; doi:10.1371/journal.pone.0296672)
Supplement: S2 Table — (PDF) [file pone.0296672.s006.pdf]

			Tet1	Tet2	Pha	Het
**Background**	**Transcripts**	**Mean**	141,2	82,0	8,3	244,9
		**Median**	127	61	5	226
	**Genes**	**Mean**	125,6	71,4	5,0	226,4
		**Median**	115	56	4	214
**Singles**	**Transcripts**	**Mean**	171,4	254,5	7,5	338,1
		**Median**	162	226	6	328
	**Genes**	**Mean**	147,6	194,3	4,7	305,8
		**Median**	143	177	4	302
**Doubles**	**Transcripts**	**Mean**	220,7	365,3	9,1	399,7
		**Median**	211	336	7	423
	**Genes**	**Mean**	183,6	262,8	5,7	358,4
		**Median**	175	254	5	384
**Clusters**	**Transcripts**	**Mean**	243,2	606,1	17,2	496,7
		**Median**	223	518	10	339
	**Genes**	**Mean**	197,4	392,1	8,4	407,3
		**Median**	193	355	6	306
**Precision**			**0,22**	**0,73**	**0,17**	**0,31**
